# Macrophage migration inhibitory factor is an early marker of severe acute pancreatitis based on the revised Atlanta classification

**DOI:** 10.1186/s12876-020-01598-0

**Published:** 2021-01-22

**Authors:** Dingcheng Shen, Caixi Tang, Shuai Zhu, Gengwen Huang

**Affiliations:** 1 Department of Hepatobiliary and Pancreatosplenic Surgery, Zhuzhou Central Hospital, Zhuzhou, 412007 Hunan Province China; 2 Department of Pancreatic Surgery, General Surgery, Xiangya Hospital, Central South University, 87 Xiangya Rd, Changsha, 410008 Hunan Province China

**Keywords:** Macrophage migration inhibitory factor, Severe acute pancreatitis, Prediction, Revised Atlanta classification

## Abstract

**Background:**

Various serum markers for early identification of severe acute pancreatitis (SAP) have been studied. Serum macrophage migration inhibitory factor (MIF) was reported to be correlated with severity of acute pancreatitis (AP) based on the 1992 Atlanta classification. However, MIF has never been proven to be predictive of disease severity based on the revised Atlanta classification (RAC). The potential predictive value of MIF needs to be further validated.

**Methods:**

Consecutive patients with AP within 48 h after symptom onset and 10 healthy control volunteers were enrolled prospectively. Serum MIF levels were measured by enzyme-linked immunosorbent assay (ELISA). The predictive value of MIF, clinical scores and other serum markers were determined.

**Results:**

Among 143 patients with AP, there were 52 (36.4%), 65 (45.5%) and 26 (18.1%) with mild, moderate and severe disease based on the RAC respectively. Compared with healthy volunteers, serum levels of MIF were significantly higher in AP patients, especially those with SAP (*P* < 0.001). Multivariate regression analysis indicated that increased serum MIF (cut-off 2.30 ng/ml, OR = 3.16, *P* = 0.008), IL-6 (cut-off 46.8 pg/ml, OR = 1.21, *P* = 0.043), APACHE II score (cut-off 7.5, OR = 2.57, *P* = 0.011) and BISAP score (cut-off 1.5, OR = 1.01, *P* = 0.038) were independent risk factors for predicting SAP (*P* < 0.05). By using the area under the receiver operating characteristic (ROC) curve (AUC), MIF (AUC 0.950) demonstrated more excellent discriminative power for predicting SAP than APACHE II (AUC 0.899), BISAP (AUC 0.886), and IL-6 (AUC 0.826).

**Conclusions:**

Serum MIF is a valuable early marker for predicting the severity of AP based on the RAC.

## Background

Acute pancreatitis (AP) is a common digestive disease with the potential to cause significant morbidity and mortality. The incidence of AP ranges from 5 to 30 cases per 100,000, and admissions have increased by at least 15% over the past 10 years [1, 2]. AP can be categorized into mild acute pancreatitis (MAP), moderately severe acute pancreatitis (MSAP), and severe acute pancreatitis (SAP) according to the revised Atlanta classification (RAC) [3]. The majority of cases (> 80%) is MAP, which is characterized by only interstitial changes of the pancreas without local or systemic complications, and the mortality is quite low. MSAP is characterized by local complications or transient organ failure (< 48 h), with a mortality of less than 5%. However, SAP is characterized with persistent failures of one or more organ system (respiratory, cardiovascular, or renal), and associated with significant morbidity and mortality [2–4]. Therefore, early identification of those who would progress to the severe category could allow the physicians to monitor more closely and put more medical resources.

A host of predictors, including clinical and laboratory markers (e.g., C-reactive protein and interleukins 6, 8, and 10) [5–9] and various scoring systems [10, 11], such as Acute Physiology and Chronic Health Evaluation (APACHE) II, Bedside Index for Severity in Acute Pancreatitis (BISAP), Systemic Inflammatory Response Syndrome (SIRS), have been studied to be associated with prediction of severity of AP. However, these predictors are either inconvenient to use or of limited clinical value. Early risk-stratification of AP patients remains a great challenge. There is an urgent clinical need to identify a reliable predictor of disease severity.

Macrophage migration inhibitory factor (MIF) was originally described in 1966 as a cytokine derived from activated T lymphocytes. It prevented random macrophage migration at the site of inflammation [12]. It belonged to the group of pro-inflammatory cytokines and was considered as a crucial upstream regulator of the innate immune reaction [13, 14]. Previous studies [15–19] reported that increased MIF was associated with several diseases such as rheumatoid arthritis (RA) [15], disseminated intravascular coagulation (DIC) [16], acute respiratory distress syndrome (ARDS) [17], sepsis [18] and other critical illnesses [19]. As for AP, elevations of serum and ascitic MIF levels have been demonstrated in rats with experimental pancreatitis and prophylactic administration of anti-MIF antibody significantly improved the survival rate of the rats [20]. Serum MIF levels were also found to be higher in patients with SAP or with pancreatic necrosis (PN) compared with those with mild attack or without PN [21]. However, to the best of our knowledge, serum MIF has never been proven to be predictive of severity of AP based on the RAC [22, 23]. Therefore, the aim of the present study is to validate the hypothesis that serum MIF might serve as an early marker to predict the severity of AP defined by the RAC.

The study was designed, conducted and reported according to STROBE guidance [24] for observational studies.

## Methods

### Patients

This study was conducted according to the guidelines laid down in the Declaration of Helsinki and all procedures involving human subjects were approved by the Ethics Committee of Xiangya Hospital, Central South University (a tertiary referral center with an average of 300 admissions with AP annually), China (reference: 2019010008). Written informed consent was obtained from all subjects or their representatives for the study participation. Between June 2019 and June 2020, a consecutive cohort of 143 patients diagnosed with AP were recruited and 10 healthy volunteers matched with sex and age were included as control subjects. All patients included in this study were admitted to the hospital within 48 h of onset of symptoms. Inclusion criterion was: first episode of AP as defined by AGA guideline [1]. Exclusion criteria were: age below 18 or over 80 years; advanced chronic respiratory, renal, heart and immune diseases. All patients received standard conservative treatment according to the latest international guidelines [1, 25, 26], Patients with organ failure were treated with organ-specific support as needed, including mechanical ventilation, continuous renal replacement therapy, vasoactive agents, and others. Step-up surgical interventions were generally performed in cases of infected pancreatic necrosis (IPN) and, if possible, postponed at least 3 ~ 4 weeks since disease onset [27].

### Definitions

The diagnosis and classification of AP were based on the RAC and AGA guideline [1, 3]. The criteria for organ failure (OF) was defined for 3 organ systems (respiratory, cardiovascular, or renal) on the basis of the worst measurement over a 24-h period. Respiratory failure: PaO2/FiO2 ≤ 300 mmHg (≤ 40 kPa) or a need for mechanical ventilation; cardiovascular failure: circulatory systolic blood pressure < 90 mmHg, despite adequate fluid resuscitation or need for inotropic agent; renal failure: creatinine ≥ 171 μmol/L (≥ 2.0 mg/dL) or a need for hemofiltration or hemodialysis. Persistent OF was defined as OF in the same organ system for 48 h or more. PN was characterized by presence of pancreatic parenchymal necrosis more than 30% on intravenous contrast enhanced CT performed after 72-h of attack, and IPN was defined as a positive culture of (peri)pancreatic necrotic fluid obtained during the first drainage or necrosectomy. These definitions were all consistent with the AGA guideline on AP [1].

### Sample and data collection

Peripheral venous blood samples were obtained immediately on admission (within 48 h of onset of symptom) of each patient and each healthy volunteer. Plasma used for testing MIF was obtained after centrifugation (3000 × *g*, 10 min, 4 °C) and stored at − 80 °C for further analysis. Demographic and clinical data were recorded in an electronic database prospectively. APACHE II, BISAP and SIRS scores were calculated within 24 h of admission. Routine clinical serum markers including white blood cell (WBC), creatinine, fibrinogen, CRP, procalcitonin (PCT), IL-6, IL-10 and tumor necrosis factor-α (TNF-α) were reported by the Department of Clinical Biochemistry of Xiangya hospital. All patients were followed until discharge from the hospital or until death.

### Serum MIF assay

Serum MIF concentration was measured by a quantitative sandwich enzyme linked immunosorbent assay (ELISA) (R&D Systems, Minneapolis, MN, USA) following the recommended protocols in the supplemental manufacturer’s instructions. The range limit of detection was 0.2–16 ng/mL.

### Statistical analysis

Continuous variables were expressed using mean ± standard deviation (SD) and median ± interquartile ranges (IQR), and categorical variables were described in absolute numbers and in percentages, in the univariate analysis, the Fisher exact test, the χ2 test, and binary logistic regression analysis were used for bivariate comparisons. Then the significant variables were included in the multivariable analysis, which were performed using logistic regression analysis as we described previously [4]. Receiver operating characteristic (ROC) curves were constructed for predictive variables, and the area under the curve (AUC) with 95 per cent confidence intervals calculated, optimal cut-off values for sensitivity, specificity for each parameter were derived from the ROC curves [5]. All tests were bilateral, and P-values < 0.05 were considered statistically significant. The SPSS (22.0) was used for all analyses.

## Results

### Patient clinical characteristics

A total of 143 patients with AP (94 men and 49 women) were included in the study. Median age at admission was 47 years old (26–76). Hyperlipidemia was the most common cause (n = 77, 53.8%), followed by biliary (n = 46, 32.2%), other (n = 12, 8.4%), and alcoholic (n = 8, 5.6%). 52 (36.4%) of them had MAP, 65 (45.5%) had MSAP and 26 (18.1%) had SAP. PN and IPN occurred in 63 (44.1%) and 7 (4.9%) patients. The overall hospital mortality was 3.5% (5 of 143).

### Prediction of SAP

One hundred and forty-three patients with AP were divided into two groups according to the presence of persistent OF, ie, non-SAP group (including MAP and MSAP, n = 117) and SAP group (n = 26), and 10 healthy volunteers were chosen as control group. The demographic characteristics of the 3 groups were similar (*P* > 0.05). The non-SAP and SAP group differed significantly *(P* < 0.001) in terms of PN, mortality and length of hospital stay (Table 1).

**Table 1:** Comparison of baseline characteristics among the patients with different groups

Parameters	Control (n = 10)	non-SAP (n = 117)	SAP (n = 26)	*P* value
Age, (mean ± SD), years	41.40 ± 13.90	46.80 ± 14.74	49.69 ± 13.85	0.070
Male/Female, n	6/4	75/42	19/7	0.761
Etiology, n (%)				0.415
Hypertriglyceridemia		64 (54.8)	13 (50.0)	
Biliary		37 (31.6)	9 (34.6)	
Alcohol		6 (5.1)	2 (7.7)	
Other		10 (8.5)	2 (7.7)	
PN, n (%)		38 (32.5)	25 (96.2)	**0.001**
IPN, n (%)		1 (0.8)	6 (23.1)	0.124
Death, n (%)		0 (0.0)	5 (19.2)	**< 0.001**
Hospital stay, (mean ± SD), d		10.33 ± 5.68	25.63 ± 25.27	**< 0.001**

The bold in the table means the *P* value of these parameters were < 0.05 and considered statistically significant

*PN* Pancreatic necrosis, *IPN* Infected pancreatic necrosis, *SD* standard deviations

Categorical variables are described as N (%)

Compared with control and non-SAP group, clinical biomarkers and scores including neutrophil, BUN, Cr, D-dimer, CRP, PCT, IL-6, MIF, APACHE II, BISAP and SIRS scores were significantly higher in SAP group by the univariate analysis (Table 2).

**Table 2:** Comparison of clinical biomarkers and scores in different groups

Parameters	Control (n = 10)	Non-SAP (n = 117)	SAP (n = 26)	*P* value
WBC (× 10^9^/L)	5.52 ± 2.57	12.54 ± 4.58	14.28 ± 3.62	0.166
Neutrophil count (× 10^9^/L)	4.01 ± 2.13	10.12 ± 4.07	12.42 ± 3.21	**0.041**
Lymphocyte count (× 10^9^/L)	2.13 ± 1.04	1.66 ± 1.73	0.96 ± 0.40	0.115
NLR	3.64 ± 2.14	9.88 ± 6.32	15.76 ± 8.52	**0.003**
HCT (%)	42.33 ± 3.78	40.26 ± 6.20	37.94 ± 9.69	0.251
BUN (mmol/L)	4.67 ± 2.17	5.09 ± 3.44	11.83 ± 6.74	**< 0.001**
Cr (umol/L)	67.23 ± 18.55	80.69 ± 25.39	182.03 ± 123.13	**< 0.001**
D-dimer (mg/L)	0.32 ± 0.21	1.41 ± 1.81	3.85 ± 4.29	**0.001**
Fibrinogen (g/L)	3.21 ± 0.67	4.99 ± 1.72	5.73 ± 1.60	0.127
CRP (mg/L)	5.32 ± 4.26	151.16 ± 105.05	303.03 ± 222.40	**0.017**
PCT (ng/L)	0.36 ± 0.12	2.15 ± 5.54	6.33 ± 6.72	**0.033**
IL-6 (pg/ml)	3.27 ± 2.59	47.39 ± 59.21	183.33 ± 231.23	**< 0.001**
IL-10 (pg/ml)	8.23 ± 3.12	13.29 ± 31.78	14.41 ± 20.87	0.896
TNF-α (pg/ml)	4.24 ± 2.18	14.08 ± 8.75	17.96 ± 9.24	0.126
MIF (ng/ml)	0.51 ± 0.23	1.68 ± 2.04	6.04 ± 4.05	**< 0.001**
APACHE II		4.40 ± 3.74	12.19 ± 4.13	**< 0.001**
BISAP		1.02 ± 0.77	2.94 ± 1.29	**< 0.001**
SIRS		2.14 ± 2.09	4.50 ± 2.66	**< 0.001**

The bold in the table means the *P* value of these parameters were < 0.05 and considered statistically significant

*WBC* White blood cell, *NLR* Neutrophil–lymphocyte ratio, *HCT* Hematocrit, *BUN* Blood urea nitrogen, *Cr* Creatinine, *CRP* C-reactive protein, *PCT* procalcitonin, *IL* Interleukin, *TNF* Tumor necrosis factor, *MIF* Macrophage migration inhibitory factor, *APACHE* Acute Physiology And Chronic Health Evaluation, *BISAP* Bedside Index for Severity in Acute Pancreatitis, *SIRS* Systemic Inflammatory Response Syndrome

WBC, Neutrophil count, CRP, APACHE II, BISAP and SIRS were expressed as mean ± standard deviation (SD), other variables—as median ± interquartile ranges (IQR)

**Fig. 1:**
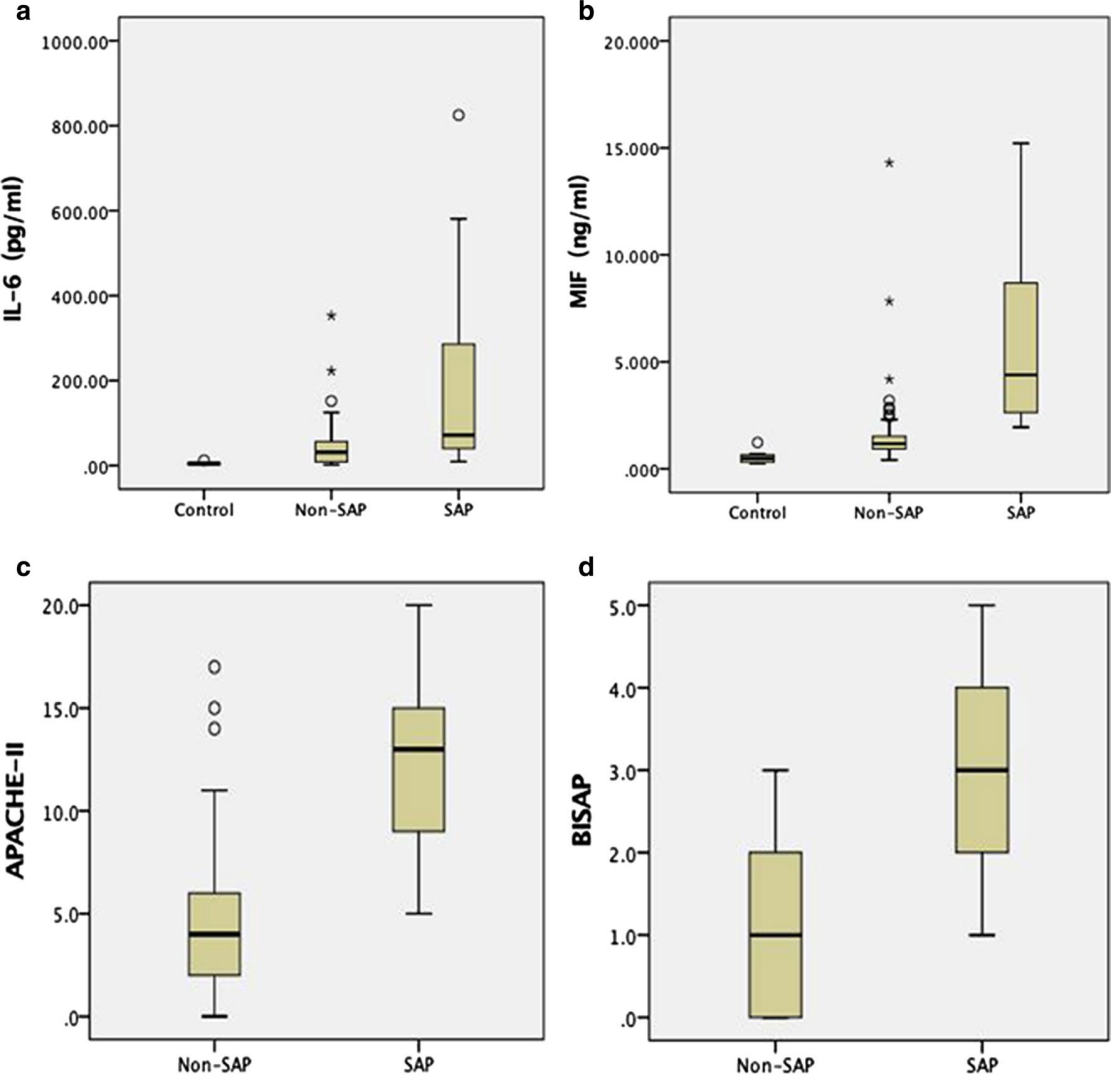
Box-whisker plots showing levels of IL-6 (**a**), MIF (**b**), APACHE II (**c**) and BISAP (**d**) score in patients with different severity of AP. Results are expressed as median, with error bars representing the IQR

The variables showing significant predictive value within univariate analysis were included in further stepwise multivariate logistic regression (Table 3). The regression analysis indicated that the increased serum IL-6 (OR = 1.21; 95% CI, 1.003–1.274; *P* = 0.043), MIF (OR = 3.16; 95% CI, 1.225–3.777; *P* = 0.008), APACHE II score (OR = 2.57; 95% CI, 1.132–3.259; *P* = 0.011) and BISAP score (OR = 1.01; 95% CI, 1.004–1.015; *P* = 0.038) were independent risk factors for predicting SAP (*P* < 0.05). Box-whisker plots showed levels of IL-6, MIF, APACHE II and BISAP score in patients with different groups (Fig. 1).

**Table 3:** Multivariate logistic regression analysis for predicting SAP

Parameters	OR	95% CI	*P* value
IL-6	1.21	1.003–1.274	**0.043**
MIF	3.16	1.225–3.777	**0.008**
APACHE II	2.57	1.132–3.259	**0.011**
BISAP	1.01	1.004–1.015	**0.038**

The bold in the table means the *P* value of these parameters were < 0.05 and considered statistically significant

*IL* Interleukin, *MIF* Macrophage migration inhibitory factor, *APACHE* Acute Physiology And Chronic Health Evaluation, *BISAP* Bedside Index for Severity in Acute Pancreatitis, *OR* Odds ratio, *CI* Confidence intervals

### Predictive value of IL-6, MIF, APACHE II and BISAP scores in SAP

**Fig. 2:**
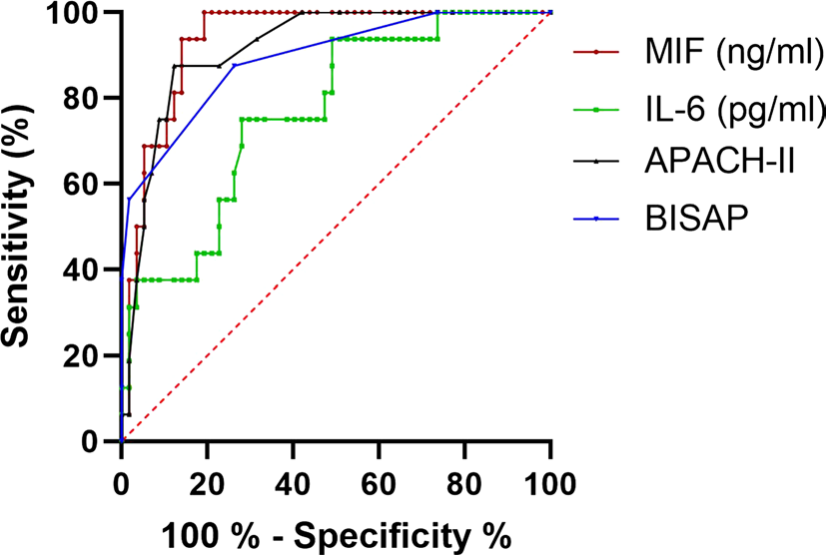
Receive operating characteristic (ROC) curve for predicting SAP by MIF, APACHE II, BISAP scores and IL-6

The ROC curves of MIF, APACHE II, BISAP scores and IL-6 were plotted for predicting SAP (Fig. 2). The AUC values were summarized in Table 4. Using the optimal cut-off value of MIF (2.30 ng/ml), it surpassed all other parameters measured in the present study, with a AUC, sensitivity and specificity of 0.950, 96.2% and 80.3%.

**Table 4:** Accuracy of the studied parameters in predicting SAP

Parameters	Cut-off	AUC (95% CI)	Sensitivity (%)	Specificity (%)
IL-6	46.8 pg/ml	0.826 (0.746–0.906)	69.2	83.8
BISAP	1.5	0.886 (0.820–0.953)	84.6	79.5
APACHE II	7.5	0.899 (0.820–0.953)	76.9	88.0
MIF	2.30 ng/ml	0.950 (0.914–0.987)	96.2	80.3

## Discussion

The present prospective cohort study has shown that serum MIF can serve as a valuable early marker to predict the severity of AP within 72 h of disease onset. It even outperformed APACHE II, BISAP and IL-6. To the best of our knowledge, this study was the first study to identify the early predictive value of serum MIF in the context of the RAC of AP.

AP is a potentially lethal disease with increasing incidence. The clinical course of AP varies greatly among patients, ranging from mild (self-limiting clinical course), through moderate (local complication or transient organ failure) to severe (persistent organ failure) disease. Accurate recognition of the severity is very crucial for the clinical decision-making [3]. For patients with predicted SAP, monitoring in the intensive care unit (ICU), early fluid resuscitation, early enteral nutrition and other interventions would be used, hopefully improving the overall prognosis [1–3]. Thus, with the purpose of early prediction of the severity of AP, a host of serum markers have been tested [6–9].

A recent large cohort study demonstrated that the level of circulating histones within 48 h after disease onset was an accurate index of disease severity, and capable of predicting persistent OF and mortality [5]. IL-6 had also been studied in detail for its role in severity stratification of AP. A meta-analysis involving 11 studies showed that serum IL-6 on day 1 to 3 after admission had a sensitivity of 81–84% and specificity between 76 and 85% for predicting SAP [28]. Jain et al. found that serum IL-6 > 160 pg/ml increased the positive predictive value of persistent SIRS from 56 to 85% and specificity from 64 to 95% for predicting SAP and then concluded that a combination of SIRS and IL-6 might be a useful and accurate predictor of severity [29]. In line with Jain’s study, this study also revealed that IL-6 was an independent risk factor (OR = 1.21), with modest sensitivity and AUC for predicting SAP. Other serum markers, including BUN, CRP, D-dimer, apolipoprotein A-I were widely investigated [29–33]. Nevertheless, none of these serum markers were highly accurate nor specific. Moreover, there were remarkable controversies among studies regarding the effectiveness of these markers.

Meanwhile, multifactorial scoring systems have also been investigated for this purpose such as BISAP and APACHE II, two of the most widely used prognostic scoring systems in AP, particularly used for research purposes. A recent prospective study involving 343 patients revealed that both BISAP (cut-off ≥ 2, sensitivity 84.1%, specificity 91.9%, AUC 0.93) and APACHE II (cut-off ≥ 7, sensitivity 92.3%, specificity 92.5%, AUC 0.98) were comparable in predicting SAP and outperformed other scoring systems [7]. The present study also proved the effectiveness of these 2 scoring systems. However, each of the existing scoring systems had its intrinsic shortcoming such as inconvenience in application in the clinical setting [10]. Physicians always wished that there would be a single marker which could outperform these scoring systems to predict the severity of AP.

MIF belongs to the group of pro-inflammatory cytokines [12]. Since reseachers discovered the inhibitory effects of MIF on T cell migration in vitro, the name “macrophage migration inhibitory factor” was defined. A series of researches demonstrated that MIF was constitutively expressed and stored within the intracellular pools. It could be released into the circulation on stimulation by proinflammatory cytokines, lipopolysaccharide (LPS), and gram-positive exotoxins without de-novo mRNA generation and protein synthesis [13, 14]. Researchers have provided evidences that MIF directly or indirectly promoted the production or expression of a large panel of proinflammatory molecules including TNF-α, IL-6, IL-8, IL-12, interferon-γ (IFN-γ), nitric oxide (NO), matrix metalloproteinase (MMP) and prostaglandin E2 (PGE2) [34, 35]. MIF has thus been shown to play an important role as a pivotal regulator of inflammation and innate immunity.

In rodent models, infusion of MIF could lead to multiple organ failure and even death, which could be reversed by anti-MIF antibodies. Elevations of MIF levels in serum and ascites have been demonstrated in experimental pancreatitis and prophylactic administration of anti-MIF antibody significantly improved the survival rate of the rats [20]. In clinical settings, serum MIF levels were elevated in patients with a severe attack of AP, particularly among those who experienced pancreatic necrosis [21]. Similar conclusions were drawn by Sakai and Dambrauskas [20, 36]. However, in these studies, the classification of the severity of AP was based on the 1992 Atlanta symposium. The biggest difference between 1992 Atlanta classification and 2012 Atlanta classification was the definition of the severe category. Therefore, the role of serum MIF in predicting the severity of AP in the context of current severity classification remains unclear.

This study had several limitations. Firstly, this cohort was conducted at a tertiary care center. Most of the patients with AP in the institution were referred from other hospitals during the varying course of AP. Among them, only a minority of patients were admitted within 48 h from disease onset. The relative small population was a limitation of the study. Secondly, in this study, we enrolled patients with AP who were admitted to the hospital within 48 h from onset of symptoms. Ideally, 48 h or even 24 h would be more persuasive to qualify an early predictor. Therefore, further larger studies enrolling a larger number of patients with AP and limiting the time to 24 h or earlier would be needed. Thirdly, a high number of cases resulting from hypertriglyceridemia as compared to other available studies, as the prevalence of hypertriglyceridemia has increased dramatically in China with unclear reasons. Therefore, the frequency of hypertriglyceridemia-induced acute pancreatitis (HTG-AP) also increased over the years.

## Conclusions

To the best of our knowledge, the present study was the first study to investigate the early predictive value of MIF on the severity of AP based on the RAC. The results showed that serum MIF was elevated in patients with AP when compared with healthy controls. Moreover, at a cutoff value of 2.30 ng/ml of MIF, SAP was predicted with a sensitivity of 96.2% and a specificity of 80.3%, indicating that elevated levels of serum MIF (AUC 0.950) was an accurate index of disease severity, and capable of predicting SAP. It even outperformed APACHE II (AUC 0.899), BISAP (AUC 0.886) and IL-6 (AUC 0.826). Given that detection of serum MIF is easily available and relatively inexpensive, serum MIF would hopefully become a potential valuable marker for early identification of patients with SAP.

## Data Availability

The datasets generated and/or analyzed in the present study are available from the corresponding author on reasonable request.

## Abbreviations

AP: Acute pancreatitis
APACHE: Acute Physiology and Chronic Health Evaluation
BISAP: Bedside Index for Severity in Acute Pancreatitis
ELISA: Enzyme-linked immunosorbent assay
MIF: Macrophage migration inhibitory factor
OF: Organ failure
RAC: Revised Atlanta classification
SAP: Severe acute pancreatitis
SIRS: Systemic Inflammatory Response Syndrome
